# Supplementary material for: Spatial variance of spring phenology in temperate deciduous forests is constrained by background climatic conditions
Source: Nat Commun. 2019 Nov 26;10:5388. doi: 10.1038/s41467-019-13365-1 (PMC6879605; doi:10.1038/s41467-019-13365-1)
Supplement: Supplementary file 1 — Supplementary Information [file 41467_2019_13365_MOESM1_ESM.pdf]

Spatial variance of spring phenology in temperate deciduous forests is constrained by background climatic conditions

Peaucelle et al.

Supplementary Figures and Tables.

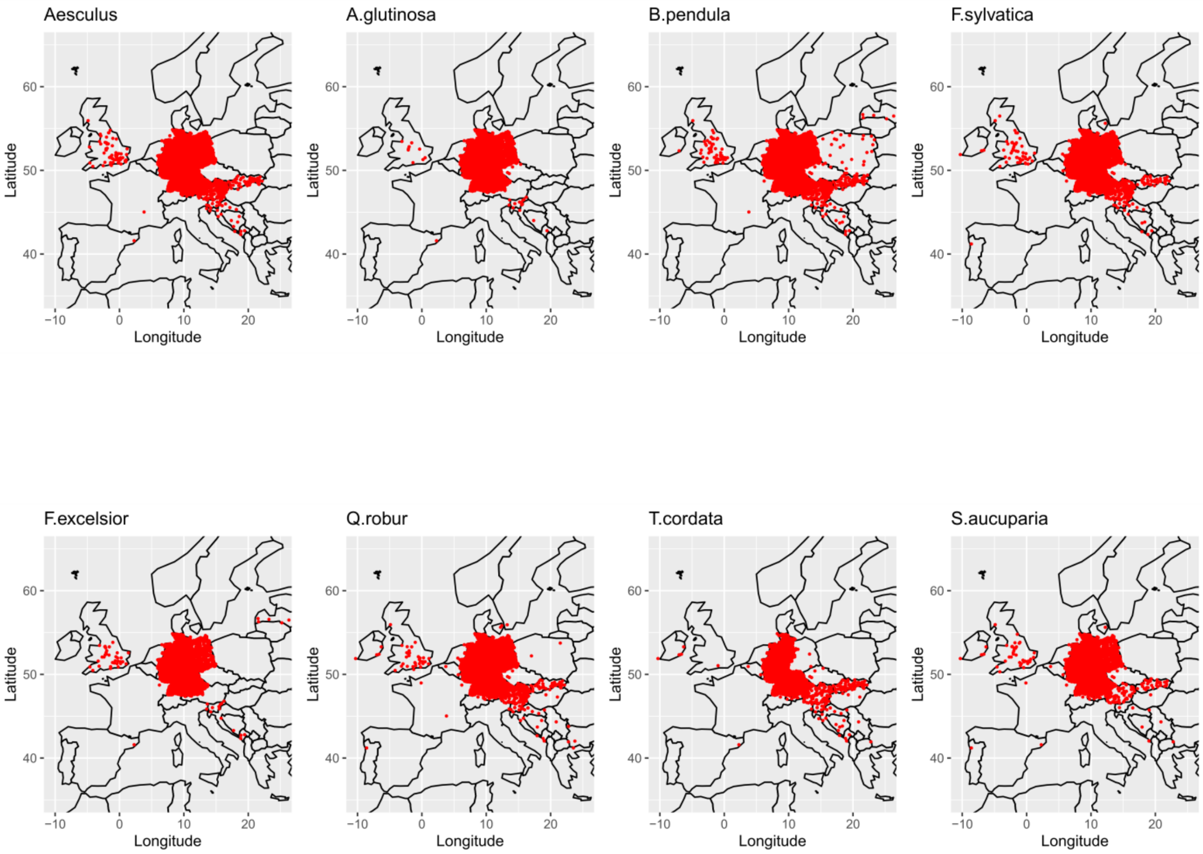

Supplementary Figure 1 | Distribution of sites for each species: *Aesculus hippocastanum*, *Alnus glutinosa*, *Betula pendula*, *Fagus sylvatica*, *Fraxinus excelsior*, *Quercus robur*, *Tilia cordata* and *Sorbus aucuparia*.

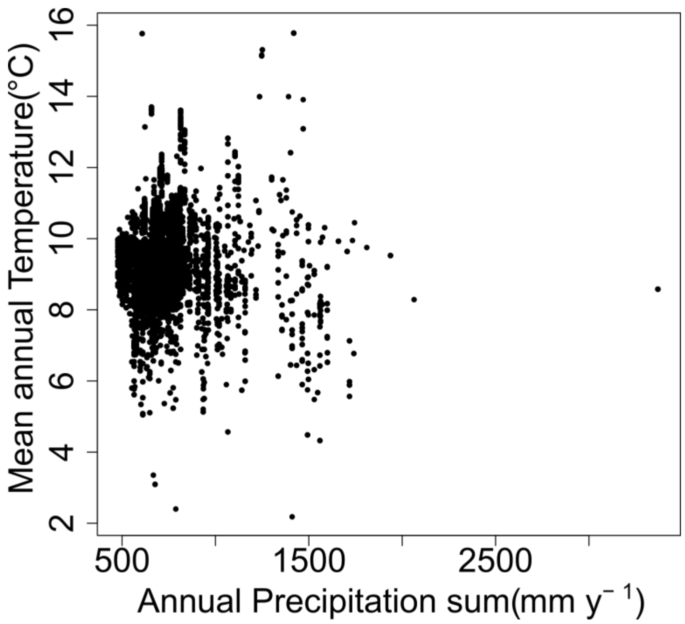

Supplementary Figure 2 | Site distribution along long-term (1970-2016) mean annual temperature and annual precipitation sum estimated with CRU-NCEP. Source data are provided as a Source Data file.

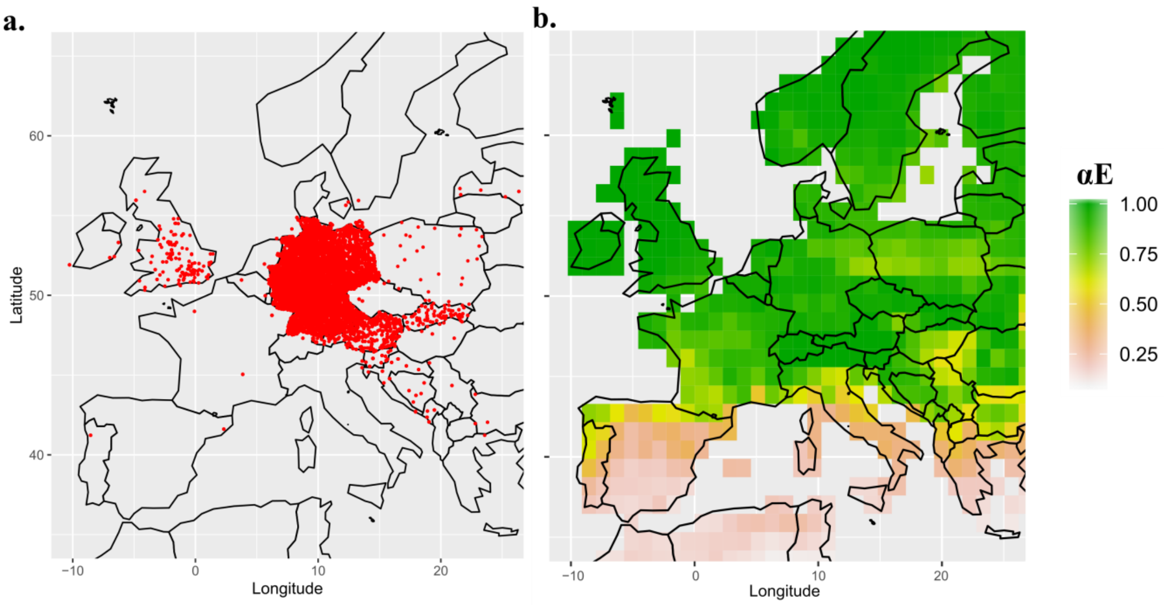

Supplementary Figure 3 | a. Spatial distribution of all sites over Europe (red dots) . **b.** Long-term average of the actual to potential evapotranspiration ratio αE.

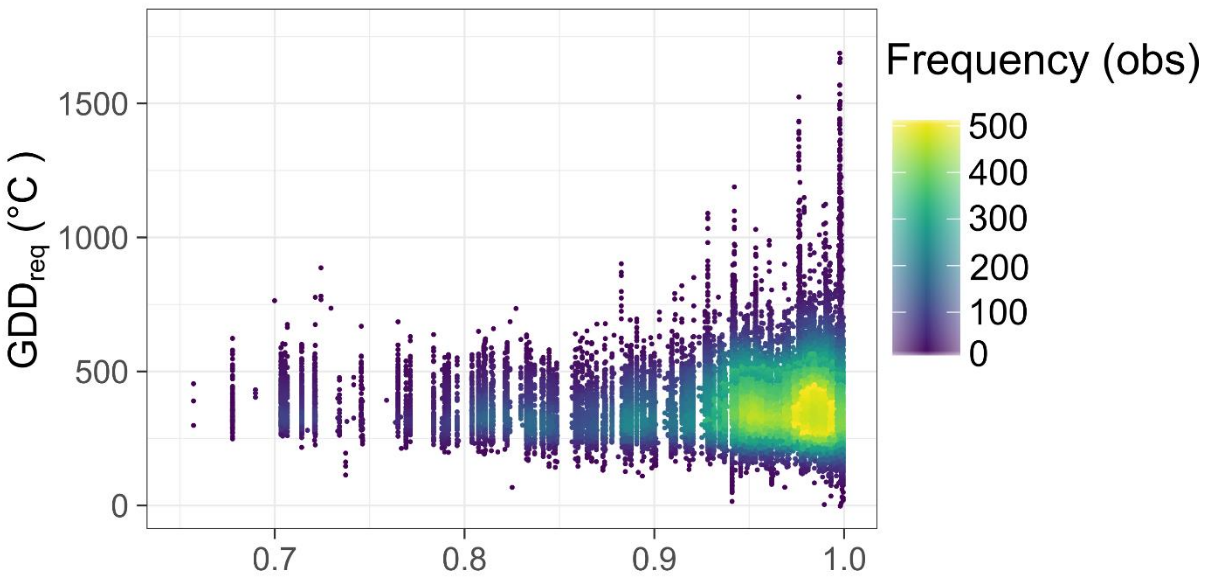

Supplementary Figure 4 | Distribution (all sites and species) of the long-term (1970-2016) heat requirement (GDD_{req}, estimated as the sum of temperatures >5 °C between 1 January and the LU date) as a function of the ratio of actual to potential evapotranspiration (αE). Low αE values indicate drought conditions during the growing season. The gradient color represents the density of observations. Source data are provided as a Source Data file.

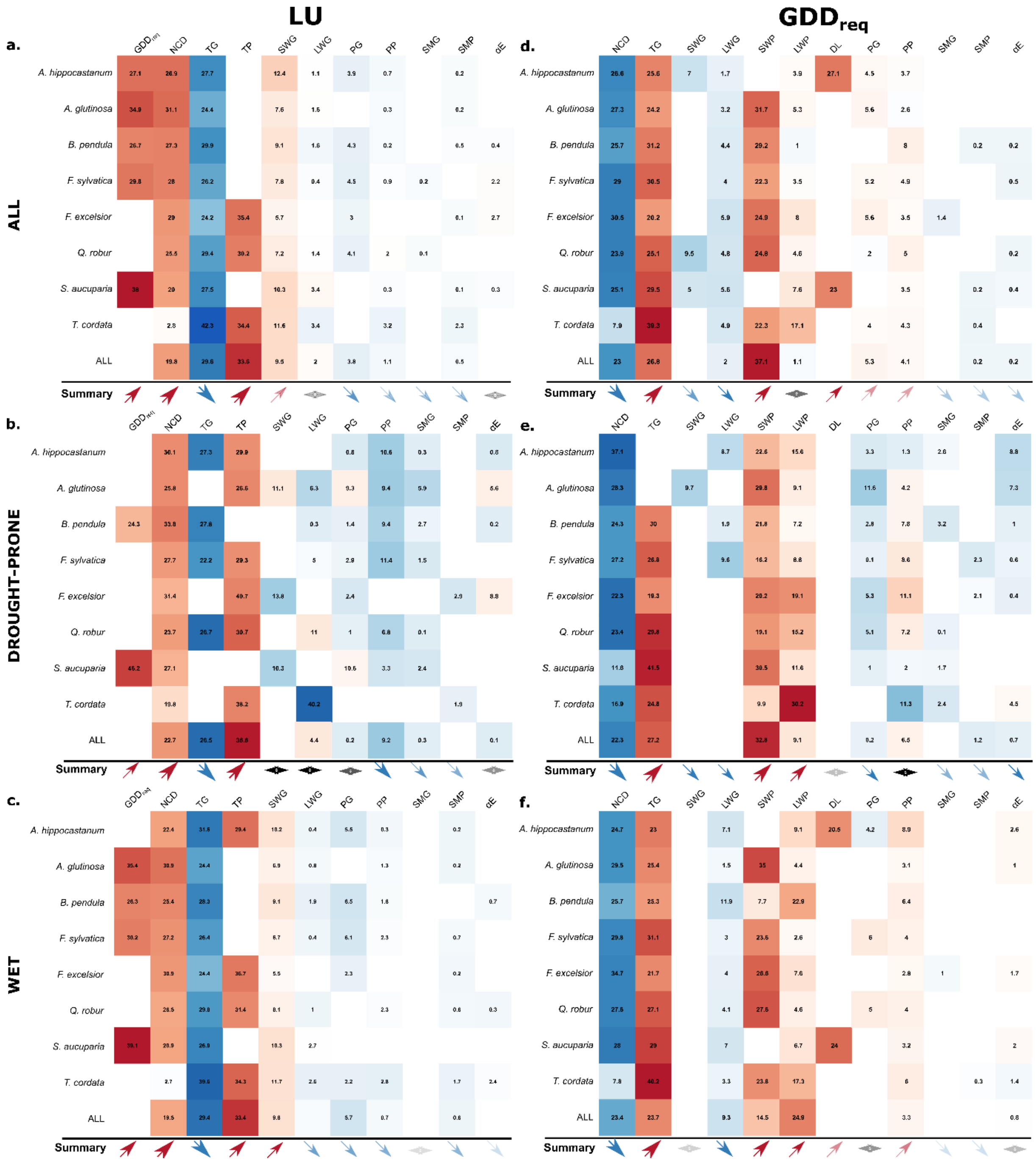

Supplementary Figure 5 | Relative importance of each variable (in percentage) in explaining leaf unfolding date (LU) and corresponding heat requirement (GDD_{req}, estimated as the sum of temperatures >5 °C between 1 January and the LU date) for each species considering a-d) all sites, b-e) only drought prone sites and c-f) wet sites. Red colors indicate a positive correlation, while blue colors indicate a negative correlation. Color intensity reflects variable relative importance. Blanks represent variables that were discarded during the predictors selection. See Supplementary Table 2 for the description of each variable. The direction and importance of correlations among species is summarized by the size and color of arrows at the bottom of each panel. A double black arrow means that the direction of the response is species dependent. Refers to Supplementary Tables 5-10 in appendix for a complete description of correlation coefficients.

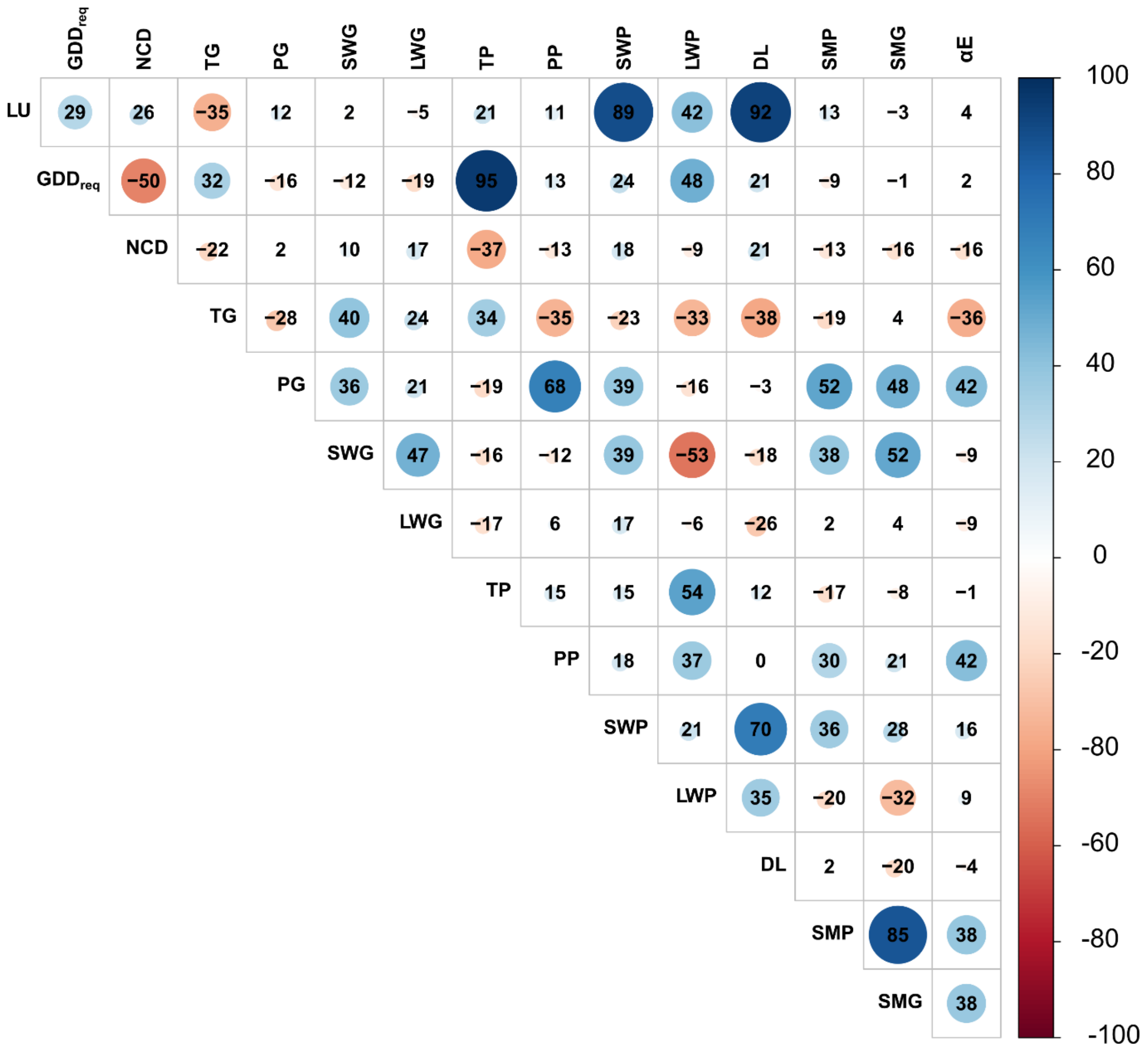

Supplementary Figure 6 | Pearson correlation coefficient (in %) between predictors. GDD_{req}, growing degree day estimated as the sum of daily temperature above 5°C from the 1 January to leaf unfolding (LU). NCD, number of chilling days estimated as the number of days between 1 November in the previous year and the LU date with temperatures between 0 and 5 °C; TG, mean growing-season temperature; TP, mean pre-season temperature; SWG, mean growing-season shortwave [visible and near infrared] radiation; LWG, mean growing-season longwave [infrared] radiation; SWP, mean pre-season shortwave [visible and near infrared] radiation; LWP, mean pre-season longwave [infrared] radiation; PG growing-season total precipitation; PP, pre-season total precipitation; DL, day length at LU date; SMP, preseason soil-moisture content; SMG, growing season soil-moisture content; αE, ratio of actual to potential evapotranspiration.

Supplementary Figure 7 | Semi-variograms of the standardized residuals of LU (as a function of site distance in km) before and after correction for spatial auto-correlation. Data (all sites) for *A.hippocastanum*, *A. glutinosa*, *B. pendula* and *F.sylvatica*.

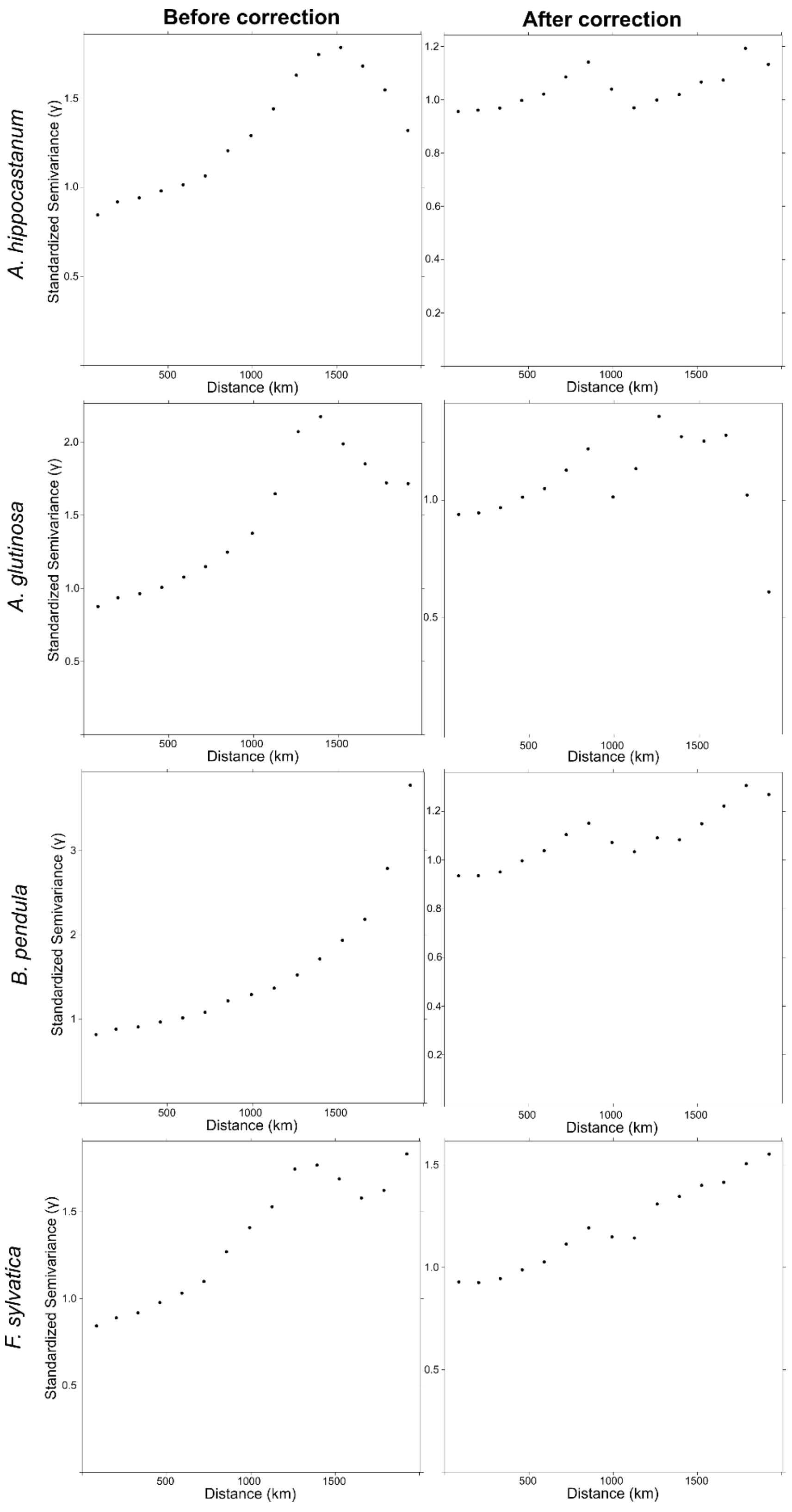

Supplementary Figure 8 | Semi-variograms of standardized residuals for LU (as a function of site distance in km) before and after correction for spatial auto-correlation. Data (all sites) for *F.excelsior*, *Q. robur*,*S. aucuparia* and *T.cordata*.

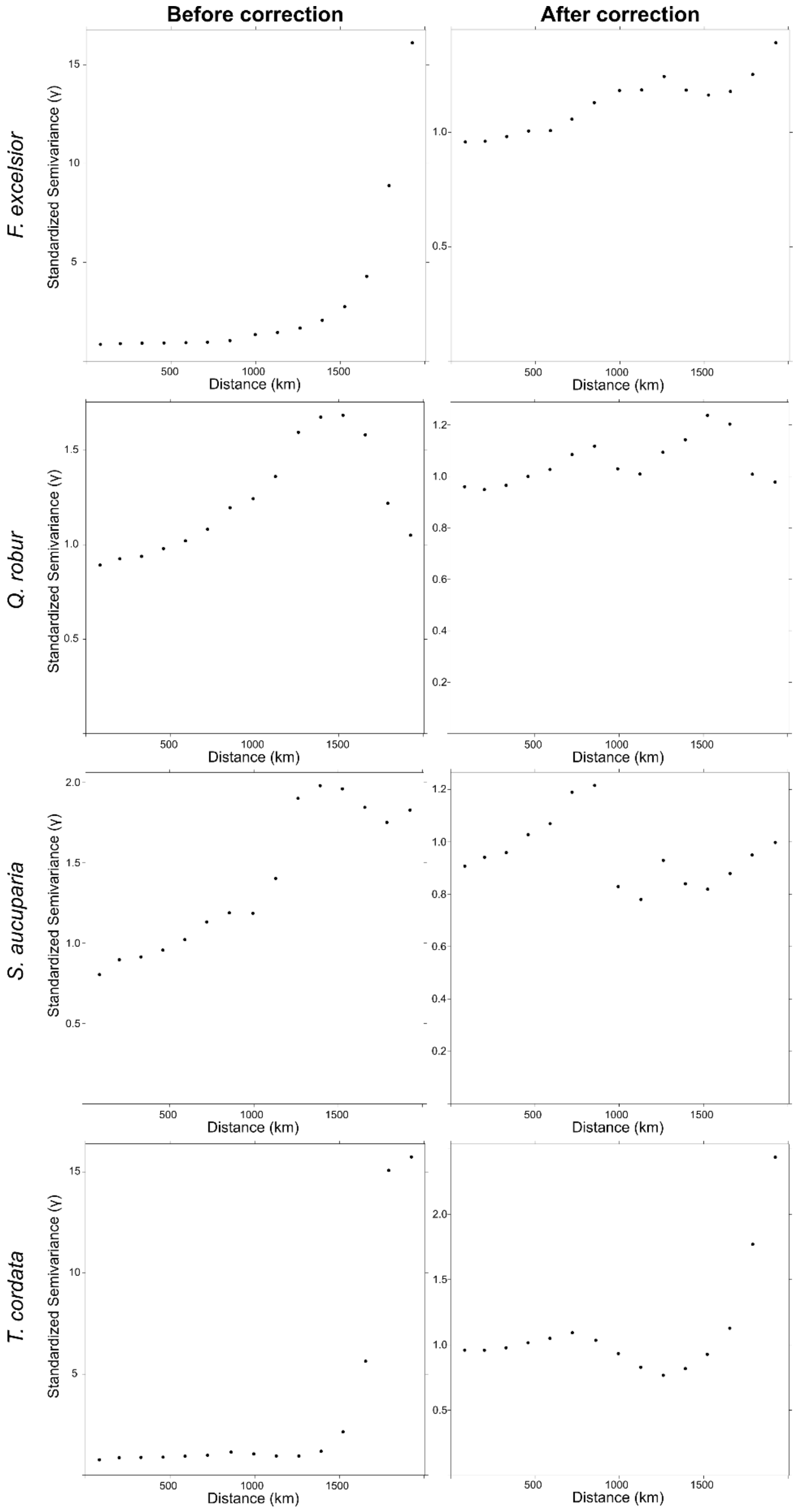

Supplementary Figure 9 | Semi-variograms of standardized residuals for LU (as a function of site distance in km) before and after correction for spatial auto-correlation. Data for all sites and all species pooled together.

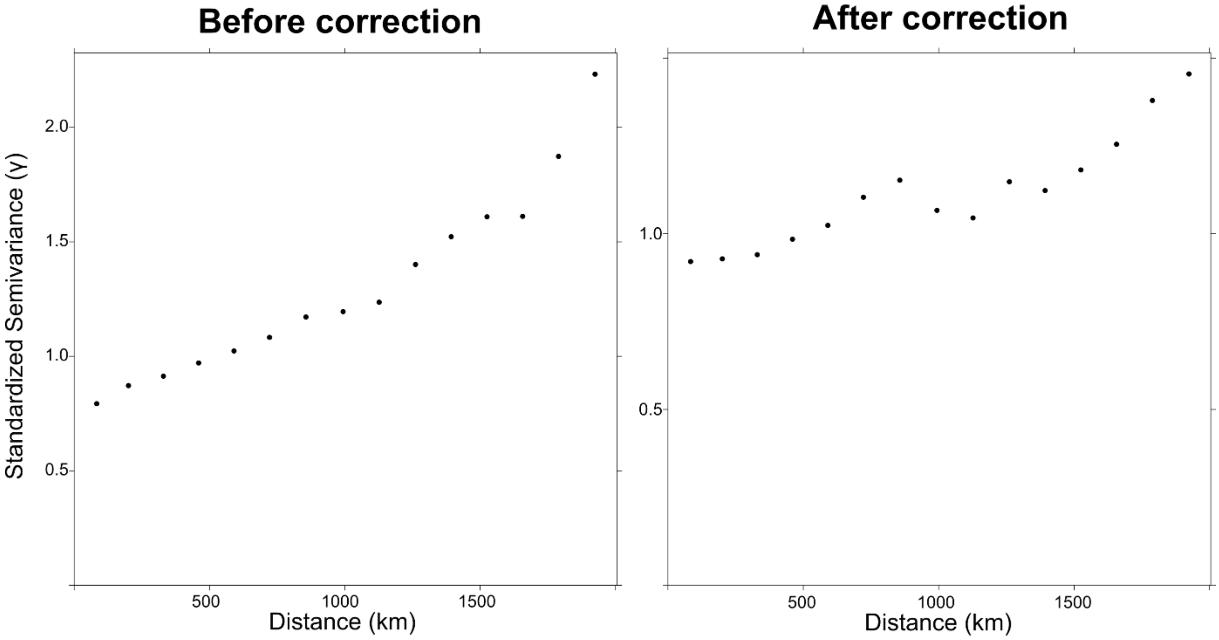

Supplementary Table 1 | Description of environmental variables and data source.

Variable	Description	Units	Data source
TG, TP	Long-term growing and preseason average of temperature	°C	CRUN-NCEP
PG, PP	Long-term growing and preseason sum of precipitation	mm	CRUN-NCEP
SWG, SWP	Long-term growing and preseason average of incoming shortwave radiations (visible and near infrared)	W m^{-2}	CRUN-NCEP
LWG; LWP	Long-term growing and preseason average of incoming longwave radiations (infrared)	W m^{-2}	CRUN-NCEP
DL	Long-term day length	h	Calculated with the geosphere[56] package
SMG,SMP	Long-term growing and preseason soil moisture between 10 and 40 cm depth	Kg m^{-2}	GLDAS https://ldas.gsfc.nasa.gov/gldas/
αE	Long-term ratio of actual to potential evapotranspiration	unitless	Calculated with the SPLASH[40] model

Supplementary Table 2 | Regression coefficients (standard deviation) and p value of generalized least square regression results for leaf unfolding dates for all sites. R^2 and RMSE represent the coefficient of determination and the root mean square error of simulated versus observed leaf unfolding dates, respectively. Bold values indicate significant result with $p < 0.1$.

	**GDD_{req}**	**NCD**	**TG**	**TP**	**SWG**	**LWG**	**PG**	**PP**	**SMG**	**SMP**	**αE**	**R^2**	**RMSE**
A. hippocastanum	**5.73 (0.09)**	**5.67 (0.1)**	**-5.8 (0.12)**		**2.6 (0.32)**	0.24 (0.24)	**-0.83 (0.28)**	-0.14 (0.17)		0.03 (0.12)	-0.01 (0.18)	0.66	5.14
P value	<1e-10	<1e-10	<1e-10		<1e-10	0.32	3.7e-3	0.39		0.78	0.95		
A. glutinosa	**6.54 (0.11)**	**5.83 (0.12)**	**-4.57 (0.15)**		**1.43 (0.36)**	0.30 (0.20)		-0.06 (0.15)		0.03 (0.15)		0.64	5.21
P value	<1e-10	<1e-10	<1e-10		7.8e-5	0.14		0.7		0.85			
B. pendula	**5.13 (0.09)**	**5.24 (0.09)**	**-5.74 (0.11)**		**1.75 (0.36)**	-0.31 (0.29)	**-0.83 (0.29)**	-0.05 (0.17)		0.09 (0.12)	0.08 (0.17)	0.55	5.21
P value	<1e-10	<1e-10	<1e-10		1.3e-6	0.28	4.7e-3	0.78		0.46	0.63		
F. sylvatica	**5.52 (0.11)**	**5.19 (0.11)**	**-4.86 (0.12)**		**1.44 (0.37)**	0.07 (0.27)	**-0.83 (0.27)**	0.18 (0.15)	-0.04 (0.16)		**0.4 (0.16)**	0.51	8.29
P value	<1e-10	<1e-10	<1e-10		8.2e-5	0.79	2.4e-3	0.24	0.78		1.4e-2		
F. excelsior		**4.6 (0.11)**	**-3.84 (0.13)**	**5.6 (0.09)**	**0.9 (0.39)**		**-0.48 (0.22)**			0.02 (0.14)	**0.4 (0.18)**	0.65	12.68
P value		<1e-10	<1e-10	<1e-10	2.1e-2		3.1e-2			0.9	1.7e-2		
Q. robur		**4.48 (0.09)**	**-5.15 (0.1)**	**5.3 (0.08)**	**1.26 (0.34)**	0.25(0.25)	**-0.73 (0.26)**	**0.35 (0.14)**	-0.02 (0.15)			0.66	8.11
P value		<1e-10	<1e-10	<1e-10	2.3e-4	0.32	4.7e-3	1.4e-2	0.89				
S. aucuparia	**8.12 (0.17)**	**4.3 (0.17)**	**-5.87 (0.15)**		**2.2 (0.44)**	**0.73 (0.33)**		0.07 (0.16)		0.03 (0.17)	0.06 (0.23)	0.5	7
P value	<1e-10	<1e-10	<1e-10		6.2e-7	2.7e-2		0.67		0.86	0.79		
T. cordata		**0.51 (0.12)**	**-7.73 (0.15)**	**6.29 (0.09)**	**2.12 (0.42)**	**-0.63 (0.36)**		**-0.58 (0.14)**		**-0.42 (0.14)**		0.68	18.83
P value		2.7e-5	<1e-10	<1e-10	4.7e-7	8e-2		2.7e-5		3.5e-3			
**ALL species**		**4.45 (0.1)**	**-6.64 (0.12)**	**7.55 (0.12)**	**2.14 (0.4)**	0.45 (0.28)	**-0.84 (0.29)**	-0.26 (0.16)		-0.1 (0.14)		0.67	6.75
P value		<1e-10	<1e-10	<1e-10	6.5e-8	0.11	3.5e-3	0.11		0.45			

Supplementary Table 3 | Regression coefficients (standard deviation) and p value of generalized least square regression results for leaf unfolding heat requirement (GDD_{req}) for all sites. R² and RMSE represent the coefficient of determination and the root mean square error of simulated versus observed leaf unfolding dates, respectively. Bold values indicate significant result with $p < 0.1$.

	NCD	TG	SWG	LWG	SWP	LWP	DL	PG	PP	SMG	SMP	αE	R2	RMSE
A. hippocastanum	**-56.5**	**54.5**	**-14.9**	-3.6		**8.3**	**57.6**	**9.5**	**7.8**				0.67	46.68
	**(1)**	**(1.1)**	**(3.1)**	(2.3)		**(1.4)**	**(1.3)**	**(2.8)**	**(1.6)**					
P value	<1e-10	<1e-10	1.6e-6	0.12		9.9e-9	<1e-10	5.5e-4	1.9e-6					
A. glutinosa	**-54.4**	**48.1**		**-6.5**	**63**	**10.6**		**11.1**	**5.2**				0.49	98.9
	**(1.3)**	**(1.4)**		**(2.3)**	**(1.6)**	**(1.9)**		**(2.8)**	**(1.8)**					
P value	<1e-10	<1e-10		5.9e-3	<1e-10	1.3e-8		9.2e-5	2.9e-3					
B. pendula	**-47**	**56.9**		**-8**	**53.3**	1.8			**14.6**		-0.4	-0.4	0.33	73.74
	**(0.9)**	**(1)**		**(2.5)**	**(1.2)**	(1.4)			**(1.2)**		(1)	(1.4)		
P value	<1e-10	<1e-10		1.5e-3	<1e-10	0.18			<1e-10		0.65	0.75		
F. sylvatica	**-60.4**	**63.5**		**-8.4**	**46.5**	**7.2**		**10.9**	**10.1**			-1.1	0.44	83.44
	**(1.2)**	**(1.2)**		**(3)**	**(1.5)**	**(1.7)**		**(2.9)**	**(1.6)**			(1.8)		
P value	<1e-10	<1e-10		5.2e-3	<1e-10	2.1e-5		1.7e-4	9.2e-10			0.55		
F. excelsior	**-72.8**	**48.3**		**-14**	**59.4**	**19.2**		**13.3**	**8.4**	**-3.3**			0.54	130.41
	**(1.5)**	**(1.5)**		**(2.7)**	**(2.1)**	**(2.4)**		**(3)**	**(1.8)**	**(1.9)**				
P value	<1e-10	<1e-10		1.7e-7	<1e-10	<1e-10		1.2e-5	6.4e-6	8.6e-2				
Q. robur	**-61**	**63.9**	**-24.3**	**-12.3**	**63.2**	**11.6**		5	**12.8**			-0.6	0.55	75.32
	**(1.2)**	**(1.3)**	**(4.4)**	**(3.2)**	**(1.8)**	**(2)**		(3.4)	**(1.8)**			(1.9)		
P value	<1e-10	<1e-10	3.4e-8	9.8e-3	<1e-10	3.6e-9		0.14	<1e-10			0.77		
S. aucuparia	**-59.6**	**70.1**	**-11.8**	**-13.3**		**18.1**	**54.7**		**8.2**		-0.5	1.1	0.73	72.56
	**(1.8)**	**(1.7)**	**(4.8)**	**(3.5)**		**(2.6)**	**(1.6)**		**(2)**		(2)	(2.8)		
P value	<1e-10	<1e-10	1.4e-2	1.4e-4		<1e-10	<1e-10		4.3e-5		0.79	0.71		
T. cordata	**-16.8**	**83.9**		**-10.4**	**47.5**	**36.4**		**8.6**	**9.1**		-0.8		0.63	82.25
	**(1.2)**	**(1.2)**		**(3.6)**	**(1.4)**	**(1.5)**		**(2.6)**	**(1.5)**		(1.4)			
P value	<1e-10	<1e-10		3.7e-3	<1e-10	<1e-10		9.2e-4	7.3e-10		0.56			
**ALL species**	**-51.8**	**60.4**		**-4.6**	**83.6**	2.5		**12**	**9.3**		-0.4	-0.5	0.48	93.94
	**(1)**	**(1)**		**(2.5)**	**(1.6)**	(1.8)		**(2.6)**	**(1.5)**		(1.1)	(1.4)		
P value	<1e-10	<1e-10		6.2e-2	<1e-10	0.15		2.5e-6	7.7e-10		0.72	0.73		

Supplementary Table 4 | Regression coefficients (standard deviation) and p value of generalized least square regression results for leaf unfolding dates for drought-prone sites. R^2 and RMSE represent the coefficient of determination and the root mean square error of simulated versus observed leaf unfolding dates, respectively. Bold values indicate significant result with $p < 0.1$.

	**GDD_{req}**	**NCD**	**TG**	**TP**	**SWG**	**LWG**	**PG**	**PP**	**SMG**	**SMP**	**αE**	**R^2**	**RMSE**
A. hippocastanum		**4.2 (0.2)**	**-3.8 (0.4)**	**4.2 (0.1)**			-0.1 (0.3)	**-1.5 (0.3)**	0 (0.3)		-0.1 (0.2)	0.54	9.63
P value		<1e-10	<1e-10	<1e-10			0.69	1.8e-8	0.87		0.63		
A. glutinosa		**4.2 (0.2)**		**4.4 (0.2)**	**1.8 (0.3)**	**-1 (0.3)**	**1.5 (0.3)**	**-1.5 (0.3)**	**-1 (0.2)**		**0.9 (0.2)**	0.63	4.61
P value		<1e-10		<1e-10	<1e-10	1.0e-4	2.6e-7	1.4e-8	1.5e-6		1.7e-5		
B. pendula	**3.3 (0.1)**	**4.5 (0.2)**	**-3.7 (0.3)**			0 (0.6)	0.2 (0.3)	**-1.3 (0.3)**	0.4 (0.2)		0 (0.2)	0.55	9.1
P value	<1e-10	<1e-10	<1e-10			0.95	0.51	7.1e-7	0.13		0.9		
F. sylvatica		**3.9 (0.2)**	**-3.2 (0.3)**	**4.2 (0.2)**		0.7 (0.5)	0.4 (0.3)	**-1.6 (0.2)**	-0.2 (0.2)			0.58	4.66
P value		<1e-10	<1e-10	<1e-10		0.19	0.2	1.4e-10	0.36				
F. excelsior		**3.4 (0.2)**		**4.4 (0.2)**	**-1.5 (0.8)**		-0.3 (0.5)			-0.3 (0.3)	**1 (0.3)**	0.67	5.5
P value		<1e-10		<1e-10	4.9e-2		0.58			0.27	1.5e-3		
Q. robur		**3.9 (0.2)**	**-4.4 (0.3)**	**5.1 (0.1)**		**1.8 (0.5)**	0.2 (0.3)	**-1.1 (0.2)**	0 (0.2)			0.72	6.53
P value		<1e-10	<1e-10	<1e-10		6.7e-4	0.56	2.9e-7	0.96				
S. aucuparia	**5.8 (0.2)**	**3.4 (0.3)**			-1.3 (0.9)		**1.3 (0.5)**	-0.4 (0.3)	-0.3 (0.3)			0.33	6.92
P value	<1e-10	<1e-10			0.17		3.5e-3	0.15	0.3				
T. cordata		**2.3 (0.7)**		**4.4 (0.3)**		**-4.6 (1.1)**				0.2 (0.7)		0.55	6.13
P value		5.8e-4		<1e-10		3.4e-5				0.75			
**ALL species**		**4 (0.2)**	**-4.7 (0.3)**	**6.5 (0.2)**		0.8 (0.6)	0 (0.3)	**-1.6 (0.3)**	0.1 (0.2)		0 (0.2)	0.68	8.4
P value		<1e-10	<1e-10	<1e-10		0.16	0.9	6.1e-10	0.8		0.95		

Supplementary Table 5 | Regression coefficients (standard deviation) and p value of generalized least square regression results for leaf unfolding heat requirement (GDD_{req}) for drought-prone sites. R^2 and RMSE represent the coefficient of determination and the root mean square error of simulated versus observed leaf unfolding dates, respectively. Bold values indicate significant result with $p < 0.1$.

	**NCD**	**TG**	**SWG**	**LWG**	**SWP**	**LWP**	**PG**	**PP**	**SMG**	**SMP**	**αE**	**R^2**	**RMSe**
A. hippocastanum	**-48 (1.8)**			**-11.3 (1.6)**	**29.2 (1.4)**	**20.2 (1.5)**	**-4.2 (1.6)**	-1.7 (1.7)	**3.4 (1.2)**		**-11.3 (1.2)**	0.64	34.37
P value	<1e-10			<1e-10	<1e-10	<1e-10	9e-3	0.33	7.2e-3		<1e-10		
A. glutinosa	**-41.9 (1.8)**		**-14.4 (2.1)**		**44.1 (2.4)**	**13.5 (2.4)**	**-17.1 (2.4)**	**6.3 (2.5)**			**-10.8 (1.8)**	0.63	40.13
P value	<1e-10		<1e-10		<1e-10	1.8e-8	<1e-10	1.2e-2			2.6e-9		
B. pendula	**-33.3 (1.7)**	**41.1 (2.6)**		-2.6 (5.5)	**29.8 (1.6)**	**9.8 (2.1)**	-3.8 (2.6)	**10.7 (2.2)**	**-4.4 (2.1)**		-1.3 (2.1)	0.27	82.09
P value	<1e-10	<1e-10		0.64	<1e-10	5.0e-6	0.14	1.0e-6	4.1e-2		0.53		
F. sylvatica	**-48.9 (1.9)**	**48.2 (2.9)**		**-17.2 (5.7)**	**29.2 (1.9)**	**15.9 (2.5)**	0.1 (3.3)	**15.4 (2.5)**		**-4.1 (2)**	1.1 (2.2)	0.58	88.49
P value	<1e-10	<1e-10		2.8e-3	<1e-10	3.5e-10	0.98	1.0e-9		4.6e-2	0.63		
F. excelsior	**-44.6 (2.6)**	**38.8 (4.5)**			**40.6 (3.1)**	**38.2 (4.2)**	**-10.7 (4.8)**	**22.3 (3.4)**		**4.3 (2.7)**	-0.9 (2.8)	0.52	215.3
P value	<1e-10	<1e-10			<1e-10	<1e-10	2.6e-2	1.1e-10		0.11	0.76		
Q. robur	**-44.7 (2.2)**	**56.9 (2.8)**			**36.5 (2.8)**	**29.1 (3.2)**	**-9.7 (2.8)**	**13.8 (2.4)**	0.2 (2.1)		0 (2)	0.48	97.49
P value	<1e-10	<1e-10			<1e-10	<1e-10	5.5e-4	1.9e-8	0.94		0.98		
S. aucuparia	**-16.6 (1.9)**	**59.6 (3.1)**			**43.8 (1.7)**	**16.7 (2.8)**	-1.5 (2.8)	2.9 (2.4)	2.5 (1.9)			0.48	74.38
P value	<1e-10	<1e-10			<1e-10	4.3e-9	0.60	0.22	0.19				
T. cordata	**-34.3 (5)**	**50.2 (5)**			**20 (3.1)**	**61.2 (4.1)**		**-22.9 (3)**	-4.9 (3.3)		**9.1 (3.5)**	0.72	42.06
P value	<1e-10	<1e-10			5.1e-10	<1e-10		<1e-10	0.14		1.0e-2		
**ALL species**	**-39.4 (1.8)**	**48.2 (2.4)**			**58.1 (2.2)**	**16 (2.7)**	-0.4 (2.6)	**11.5 (2.2)**		-2.2 (1.7)	-1.3 (1.9)	0.56	150.95
P value	<1e-10	<1e-10			<1e-10	4.5e-9	0.88	2.2e-7		0.21	0.50		

Supplementary Table 6 | Regression coefficients (standard deviation) and p value of generalized least square regression results for leaf unfolding dates for wet sites. R^2 and RMSE represent the coefficient of determination and the root mean square error of simulated versus observed leaf unfolding dates, respectively. Bold values indicate significant result with $p < 0.1$.

	**GDD_{req}**	**NCD**	**TG**	**TP**	**SWG**	**LWG**	**PG**	**PP**	**SMP**	**αE**	**R2**	**RMSE**
A. hippocastanum		**4.6 (0.1)**	**-6.5 (0.1)**	**6 (0.1)**	**2.1 (0.4)**	-0.1 (0.3)	**-1.1 (0.3)**	0.1 (0.2)	0 (0.2)		0.5	7.82
P value		<1e-10	<1e-10	<1e-10	2.2e-6	0.8	1.1e-3	0.78	0.84			
A. glutinosa	**6.8 (0.1)**	**5.9 (0.1)**	**-4.7 (0.2)**		**1.3 (0.5)**	0.2 (0.3)		0.3 (0.2)	0 (0.2)		0.63	5.59
P value	<1e-10	<1e-10	<1e-10		3.3e-3	0.56		0.12	0.82			
B. pendula	**5.5 (0.1)**	**5.3 (0.1)**	**-5.9 (0.1)**		**1.9 (0.4)**	-0.4 (0.3)	**-1.4 (0.3)**	**0.4 (0.2)**	0 (0.2)	0.2 (0.1)	0.53	5.89
P value	<1e-10	<1e-10	<1e-10		1.1e-5	0.24	9.0e-5	5.1e-2	0.95	0.3		
F. sylvatica	**5.7 (0.1)**	**5.1 (0.1)**	**-5 (0.1)**		**1.2 (0.4)**	-0.1 (0.3)	**-1.1 (0.3)**	**0.4 (0.2)**	0.1 (0.1)		0.48	7.72
P value	<1e-10	<1e-10	<1e-10		3.0e-3	0.81	3.3e-4	8.5e-3	0.35			
F. excelsior		**4.9 (0.1)**	**-3.9 (0.1)**	**5.8 (0.1)**	**0.9 (0.4)**		-0.4 (0.2)		0 (0.2)		0.63	13.36
P value		<1e-10	<1e-10	<1e-10	4.9e-2		0.14		0.84			
Q. robur		**4.5 (0.1)**	**-5 (0.1)**	**5.3 (0.1)**	**1.4 (0.4)**	-0.2 (0.3)		**0.4 (0.1)**	0.1 (0.1)	0.1 (0.1)	0.66	5.15
P value		<1e-10	<1e-10	<1e-10	2.6e-4	0.55		3.6e-3	0.48	0.71		
S. aucuparia	**8.4 (0.2)**	**4.5 (0.2)**	**-5.8 (0.2)**		**2.2 (0.5)**	0.6 (0.4)					0.52	5.42
P value	<1e-10	<1e-10	<1e-10		1.1e-4	0.13						
T. cordata		**0.5 (0.1)**	**-7.4 (0.1)**	**6.4 (0.1)**	**2.2 (0.5)**	-0.5 (0.4)	-0.4 (0.3)	**-0.5 (0.2)**	**-0.3 (0.2)**	**0.4 (0.2)**	0.66	18.93
P value		5.1e-5	<1e-10	<1e-10	2.0e-6	0.19	0.18	2.0e-3	5.6e-2	4.5e-3		
**ALL species**		**4.5 (0.1)**	**-6.7 (0.1)**	**7.7 (0.1)**	**2.2 (0.5)**	0.2 (0.3)	**-1.3 (0.3)**	0.2 (0.2)	-0.1 (0.2)		0.66	6.68
P value		<1e-10	<1e-10	<1e-10	1.803E-06	0.49	1.3e-4	0.34	0.42			

Supplementary Table 7 | Regression coefficients (standard deviation) and p value of generalized least square regression results for leaf unfolding heat requirement (GDD_{req}) for wet sites. R^2 and RMSE represent the coefficient of determination and the root mean square error of simulated versus observed leaf unfolding dates, respectively. Bold values indicate significant result with $p < 0.1$.

	**NCD**	**TG**	**LWG**	**SWP**	**LWP**	**DL**	**PG**	**PP**	**SMG**	**SMP**	**αE**	**R^2**	**RMSE**
A. hippocastanum	**-56.9 (1)**	**53 (1)**	**-16.3 (0.8)**		**21 (1.2)**	**47.3 (1.2)**	**-9.6 (1.4)**	**20.5 (1.3)**			**6 (0.9)**	0.72	45.08
P value	<1e-10	<1e-10	<1e-10		<1e-10	<1e-10	<1e-10	<1e-10			<1e-10		
A. glutinosa	**-57.1 (1.5)**	**49.1 (1.6)**	-2.9 (3)	**67.7 (1.9)**	**8.5 (2.2)**			**6 (1.7)**			1.9 (1.8)	0.52	73.75
P value	<1e-10	<1e-10	0.33	<1e-10	1.1e-4			2.8e-4			0.28		
B. pendula	**-45.2 (0.9)**	**44.5 (1)**	**-21 (0.8)**	**13.5 (0.9)**	**40.3 (0.9)**			**11.3 (0.9)**				0.7	46.86
P value	<1e-10	<1e-10	<1e-10	<1e-10	<1e-10			<1e-10					
F. sylvatica	**-62.2 (1.4)**	**65 (1.3)**	**-6.2 (3.4)**	**49.2 (1.8)**	**5.4 (2)**		**12.5 (3.4)**	**8.4 (1.9)**				0.42	89.2
P value	<1e-10	<1e-10	7.1e-2	<1e-10	7.8e-3		2.7e-4	8.6e-6					
F. excelsior	**-78.1 (1.7)**	**48.9 (1.8)**	**-9 (3.5)**	**59.9 (2.3)**	**17.2 (2.7)**			**6.2 (1.8)**	-2.3 (2.8)		**3.7 (2.1)**	0.59	165.72
P value	<1e-10	<1e-10	1.1e-2	<1e-10	1.5e-10			4.7e-4	0.42		7.4e-2		
Q. robur	**-64.1 (1.4)**	**63 (1.4)**	**-9.6 (3.6)**	**64.2 (2.2)**	**10.7 (2.3)**		**11.7 (3.6)**	**9.3 (2)**				0.38	87.38
P value	<1e-10	<1e-10	8.4e-3	<1e-10	4.6e-3		1.4e-3	5.4e-6					
S. aucuparia	**-67.4 (2.3)**	**69.8 (1.9)**	**-16.9 (5)**		**16.2 (3.4)**	**57.6 (2.1)**		**7.7 (2.3)**			**4.9 (2.5)**	0.78	80.06
P value	<1e-10	<1e-10	7.8e-4		2.0e-6	<1e-10		8.4e-4			4.9e-2		
T. cordata	**-15.5 (1.1)**	**80.4 (1.2)**	**-6.6 (3.5)**	**47.7 (1.4)**	**34.6 (1.6)**			**11.9 (1.3)**		0.5 (1.5)	**-2.8 (1.5)**	0.66	96.62
P value	<1e-10	<1e-10	5.7e-3	<1e-10	<1e-10			<1e-10		0.74	5.6e-2		
**ALL species**	**-48.4 (0.9)**	**49 (1)**	**-19.2 (0.8)**	**29.9 (1.1)**	**51.4 (1.1)**			**6.9 (1)**			**1.7 (1)**	0.73	72.87
P value	<1e-10	<1e-10	<1e-10	<1e-10	0			<1e-10			9.5e-2		

Supplementary Table 8 | Coefficients, RMSE and R^2 of the elastic-net regressions performed against leaf unfolding date observations for each species. Alpha and lambda are the two regularization coefficients of the elastic net regression. VIF selection and spatial auto-correlation corrections were not applied yet here.

**Species**	**Model coefficients**											**Regularization coefficients**			
	**GDD_{req}**	**NCD**	**TG**	**PG**	**SWG**	**LWG**	**TP**	**PP**	**SMP**	**SMG**	**αE**	**lambda**	**alpha**	**RMSE**	**R^2**
A. hippocastanum	5.37	5.43	-5.84	0.48	3.08	1.06	0.00	-1.50	0.23	-0.19	-0.25	0.13	0.75	4.48	0.72
A. glutinosa	5.24	5.27	-4.63	0.00	2.48	0.41	0.76	-0.78	0.05	0.00	0.00	0.25	0.55	4.98	0.67
B. pendula	4.33	4.53	-5.39	0.85	2.64	0.52	0.00	-1.47	0.83	-0.95	-0.41	0.14	0.35	4.36	0.67
F. sylvatica	2.60	3.98	-4.81	0.49	2.09	0.42	2.10	-1.05	0.43	-0.36	-0.07	0.19	0.15	4.16	0.59
F. excelsior	1.06	4.39	-3.80	0.03	1.57	0.00	3.94	0.00	0.07	0.00	0.55	0.18	0.75	4.22	0.69
Q. robur	2.18	4.80	-5.06	0.08	2.46	0.49	3.08	-0.63	0.24	-0.07	0.00	0.19	0.35	4.13	0.73
T. cordata	5.45	4.33	-5.03	0.00	2.46	1.32	0.00	-0.19	0.29	-0.37	-0.09	0.21	0.30	4.65	0.62
S. aucuparia	3.92	2.51	-6.38	0.00	2.41	-0.52	1.40	-0.90	0.05	0.00	0.00	0.16	0.65	3.74	0.76
**ALL species**	7.47	6.27	-5.72	0.36	3.00	0.96	1.39	-1.05	0.13	0.00	0.00	0.12	0.60	5.02	0.77

Supplementary Table 9 | Coefficients, RMSE and R^2 of the elastic-net regressions performed against growing degree days (GDD_{req}) estimations for each species. Alpha and lambda are the two regularization coefficients of the elastic net regression. VIF selection and spatial auto-correlation corrections were not yet applied here.

Species	Model coefficients												Regularization coefficients			
	**NCD**	**TG**	**PG**	**SWG**	**LWG**	**PP**	**SWP**	**LWP**	**DL**	**SMP**	**SMG**	**αE**	**lambda**	**alpha**	**RMSE**	**R^2**
A. hippocastanum	-49.35	48.60	-2.96	-16.63	-14.13	11.86	17.97	18.25	24.28	0.00	0.00	0.00	4.04	0.20	42.41	0.73
A. glutinosa	-52.03	45.28	-1.13	-16.52	-7.65	9.75	23.96	17.96	26.14	0.00	0.00	0.00	4.04	0.30	48.69	0.71
B. pendula	-45.66	49.21	-5.53	-18.71	-17.08	17.71	17.58	18.30	15.80	-1.82	0.00	-1.31	3.11	0.10	40.43	0.73
F. sylvatica	-58.46	60.83	-4.98	-25.25	-18.11	14.67	16.63	18.40	18.08	0.00	0.00	-0.81	2.65	0.40	46.23	0.79
F. excelsior	-69.79	48.61	-0.78	-15.21	-7.53	9.66	20.29	22.88	29.74	-0.29	-0.02	0.00	2.25	0.85	54.89	0.74
Q. robur	-57.73	57.76	-9.50	-22.32	-17.93	14.71	26.84	23.42	23.23	0.00	0.00	-0.22	3.79	0.30	49.80	0.74
T. cordata	-76.28	60.33	-3.55	-4.83	-27.81	14.08	13.84	16.50	34.81	-0.53	0.00	5.81	11.19	0.10	63.38	0.78
S. aucuparia	-26.42	73.26	4.46	-29.16	-4.77	6.14	35.54	36.11	9.29	-4.63	3.79	0.00	1.31	0.55	35.15	0.82
**ALL species**	-63.50	60.17	-8.77	-28.22	-17.61	17.26	45.00	19.35	34.93	-1.62	0.00	-0.43	2.85	0.20	52.00	0.80

Supplementary Table 10 | Akaike index criterion (AIC) estimated for each generalized least square regression with spatial auto-correlation correction (with different residual structures: exponential, Gaussian, linear, rational quadratic and spherical) for the estimation of leaf unfolding dates and corresponding heat requirement. Minimum AIC are in bold.

**LU**	**No correction**	**Exponential**	**Gaussian**	**Linear**	**Rational quadratic**	**Spherical**
A. hippocastanum	25502	25425	25506	25506	**24613**	25506
A. glutinosa	19129	**18771**	19126	19124	18782	**18772**
B. pendula	26088	25986	26092	**25130**	25199	26092
F.sylvatica	22370	22313	21888	**21803**	21855	**21803**
F. excelsior	16971	**16612**	16667	16970	16645	**16611**
Q. robur	22461	**21897**	21961	22457	21935	**21897**
T. cordata	13417	**12701**	12749	**12701**	12718	13421
S. aucuparia	12670	**11791**	12674	12674	11856	12674
ALL species	29070	29070	27728	**27613**	27674	**27613**
**GDDreq**						
A. hippocastanum	45194	45193	45196	45196	**44634**	45196
A. glutinosa	34039	**33204**	34042	34042	33230	34042
B. pendula	46754	46712	45436	46758	**45388**	46756
F.sylvatica	41974	41822	40727	**40645**	40690	**40645**
F. excelsior	32402	32320	32403	32403	**31646**	32403
Q. robur	41858	41848	41862	41860	**41461**	41862
T. cordata	25168	**23763**	23782	25160	**23758**	25172
S. aucuparia	23035	**21802**	21874	23030	21832	**21802**
ALL species	50522	50522	50523	50524	**49094**	50522
