# Supplementary material for: Spatial variance of spring phenology in temperate deciduous forests is constrained by background climatic conditions
Source: Nat Commun. 2019 Nov 26;10:5388. doi: 10.1038/s41467-019-13365-1 (PMC6879605; doi:10.1038/s41467-019-13365-1)
Supplement: Supplementary file 3 — Reporting Summary [file 41467_2019_13365_MOESM3_ESM.pdf]

nature research

Corresponding author(s): Marc Peaucelle

Last updated by author(s): Oct 26, 2019

Reporting Summary

Nature Research wishes to improve the reproducibility of the work that we publish. This form provides structure for consistency and transparency in reporting. For further information on Nature Research policies, see Authors & Referees and the Editorial Policy Checklist .

Statistics

For all statistical analyses, confirm that the following items are present in the figure legend, table legend, main text, or Methods section.

n/a	Confirmed	
☐	☒	The exact sample size (*n*) for each experimental group/condition, given as a discrete number and unit of measurement
☒	☐	A statement on whether measurements were taken from distinct samples or whether the same sample was measured repeatedly
☐	☒	The statistical test(s) used AND whether they are one- or two-sided<br>*Only common tests should be described solely by name; describe more complex techniques in the Methods section.*
☐	☒	A description of all covariates tested
☐	☒	A description of any assumptions or corrections, such as tests of normality and adjustment for multiple comparisons
☐	☒	A full description of the statistical parameters including central tendency (e.g. means) or other basic estimates (e.g. regression coefficient) AND variation (e.g. standard deviation) or associated estimates of uncertainty (e.g. confidence intervals)
☒	☐	For null hypothesis testing, the test statistic (e.g. *F*, *t*, *r*) with confidence intervals, effect sizes, degrees of freedom and *P* value noted<br>*Give P values as exact values whenever suitable.*
☒	☐	For Bayesian analysis, information on the choice of priors and Markov chain Monte Carlo settings
☒	☐	For hierarchical and complex designs, identification of the appropriate level for tests and full reporting of outcomes
☒	☐	Estimates of effect sizes (e.g. Cohen's *d*, Pearson's *r*), indicating how they were calculated

Our web collection on statistics for biologists contains articles on many of the points above.

Software and code

Policy information about availability of computer code

Data collection	R v.3.5
Data analysis	R v.3.5

For manuscripts utilizing custom algorithms or software that are central to the research but not yet described in published literature, software must be made available to editors/reviewers. We strongly encourage code deposition in a community repository (e.g. GitHub). See the Nature Research guidelines for submitting code & software for further information.

Data

Policy information about availability of data

All manuscripts must include a data availability statement. This statement should provide the following information, where applicable:

- Accession codes, unique identifiers, or web links for publicly available datasets
- A list of figures that have associated raw data
- A description of any restrictions on data availability

All phenology data are available at http://www.pep725.eu/ and http://www.gdr2968.cnrs.fr. CRU-NCEP data can be downloaded at https://rda.ucar.edu/datasets/ds314.3/. The SPLASH model used to estimate evapotranspiration can be downloaded at https://bitbucket.org/labprentice/splash/. Soil moisture data can be downloaded at https://ldas.gsfc.nasa.gov/gldas/.

Field-specific reporting

Please select the one below that is the best fit for your research. If you are not sure, read the appropriate sections before making your selection.

☐ Life sciences ☐ Behavioural & social sciences ☒ Ecological, evolutionary & environmental sciences

For a reference copy of the document with all sections, see nature.com/documents/nr-reporting-summary-flat.pdf

Ecological, evolutionary & environmental sciences study design

All studies must disclose on these points even when the disclosure is negative.

Study description	This study assessed the long term adaptation of spring leaf unfolding of deciduous forests to background climatic condition. The study used existing datasets of leaf unfolding observations across Europe (27790 sites) for 8 dominant tree species over the period 1970-2016.
Research sample	Phenology data are available at http://www.pep725.eu/ and http://www.gdr2968.cnrs.fr
Sampling strategy	All data from the dataset were used for the following species: Aesculus hippocastanum, Alnus glutinosa, Betula pendula, Fagus sylvatica, Fraxinus excelsior, Quercus robur, Sorbus aucuparia and Tilia cordata
Data collection	Data observations follow the BBCH protocol. Only leaf unfolding data corresponding to the BBCH code 11 were used.
Timing and spatial scale	1970-2016, Europe
Data exclusions	Leaf unfolding dates outside two interquartiles around the median distribution (i.e. outside days 80-152) were excluded, which potentially represent a response to extreme events.
Reproducibility	All statistical analysis were performed with existing R packages, all data are fully accessible online.
Randomization	All data from the dataset were used, no randomization was needed for the analysis.
Blinding	In this study we were looking at the effect of environment on leaf unfolding. Blinding is irrelevant in our context.

Did the study involve field work? ☐ Yes ☒ No

Reporting for specific materials, systems and methods

We require information from authors about some types of materials, experimental systems and methods used in many studies. Here, indicate whether each material, system or method listed is relevant to your study. If you are not sure if a list item applies to your research, read the appropriate section before selecting a response.

Materials & experimental systems

n/a	Involved in the study	
☒	☐	Antibodies
☒	☐	Eukaryotic cell lines
☒	☐	Palaeontology
☒	☐	Animals and other organisms
☒	☐	Human research participants
☒	☐	Clinical data

Methods

n/a	Involved in the study	
☒	☐	ChIP-seq
☒	☐	Flow cytometry
☒	☐	MRI-based neuroimaging
